# Association of Urinary Cadmium and Antimony with Osteoporosis Risk in Postmenopausal Brazilian Women: Insights from a 20 Metal(loid) Biomonitoring Study

**DOI:** 10.3390/toxics13060489

**Published:** 2025-06-10

**Authors:** Carlos Tadashi Kunioka, Vanessa Cristina de Oliveira Souza, Bruno Alves Rocha, Fernando Barbosa Júnior, Luís Belo, Maria Conceição Manso, Márcia Carvalho

**Affiliations:** 1 FP-I3ID, FP-BHS, Fernando Pessoa University, Praça de 9 de Abril 349, 4249-004 Porto, Portugal; 36181@ufp.edu.pt (C.T.K.); cmanso@ufp.edu.pt (M.C.M.); 2 Western Paraná State University (UNIOESTE), Cascavel 85819-110, PR, Brazil; 3 Department of Clinical Analyses, Toxicology and Food Sciences, School of Pharmaceutical Sciences of Ribeirao Preto, University of Sao Paulo, Ribeirão Preto 14040-903, SP, Brazil; vcosouza@fcfrp.usp.br (V.C.d.O.S.); farmbrunorocha@gmail.com (B.A.R.); fbarbosa@fcfrp.usp.br (F.B.J.); 4 UCIBIO i4HB, Faculty of Pharmacy, University of Porto, Rua Jorge Viterbo Ferreira 228, 4050-313 Porto, Portugal; luisbelo@ff.up.pt; 5 LAQV/REQUIMTE, University of Porto, 4050-313 Porto, Portugal; 6 RISE-Health, Faculty of Health Sciences, Fernando Pessoa University, Fernando Pessoa Teaching and Culture Foundation, Rua Carlos da Maia 296, 4200-150 Porto, Portugal

**Keywords:** metals, metalloids, environmental exposure, bone mineral density, women, aging, osteoporosis

## Abstract

Osteoporosis is a major public health concern, particularly among postmenopausal women. Environmental exposure to metals has been proposed as a potential contributor to osteoporosis, but human data remain limited and inconsistent. This study investigated changes in urinary concentrations of 20 metal(loid)s in patients with osteoporosis, as well as the association of these elements with bone mineral density (BMD), in a cohort of 380 postmenopausal women aged 50–70 years from Cascavel, Paraná, Brazil. Demographic, lifestyle, and clinical data were collected, and urinary concentrations of aluminum (Al), barium (Ba), cadmium (Cd), cobalt (Co), cesium (Cs), copper (Cu), mercury (Hg), lithium (Li), manganese (Mn), molybdenum (Mo), nickel (Ni), lead (Pb), rubidium (Rb), antimony (Sb), selenium (Se), tin (Sn), strontium (Sr), thallium (Tl), uranium (U), and zinc (Zn) were measured by inductively coupled plasma mass spectrometry. BMD was assessed at the lumbar spine, femoral neck, and total hip using dual-energy X-ray absorptiometry. Osteoporosis was diagnosed in 73 participants (19.2%). Osteoporotic women had significantly higher urinary concentrations of Cd, Mn, Pb, Sb, Sn, and Zn (*p* < 0.05). Statistically significant negative correlations were observed between BMD and urinary concentrations of Al, Cd, Hg, Mn, Sb, and U. After adjustment for confounders, elevated urinary concentrations of Cd, Mn, Pb, and Sb remained independently and significantly associated with higher odds of osteoporosis, with Cd (aOR = 1.495; *p* = 0.026) and Sb (aOR = 2.059; *p* = 0.030) showing the strongest associations. In addition, women with urinary concentrations above the 90th percentile for both Cd and Sb had a significantly higher prevalence of osteoporosis compared to those with lower levels (44.4% vs. 18.0%; *p* = 0.011). Longitudinal studies are needed to confirm causality and inform prevention strategies.

## 1. Introduction

Osteoporosis is a highly prevalent osteometabolic disorder characterized by a reduction in bone mass and the deterioration of bone microarchitecture, resulting in increased bone fragility and risk of fracture [1,2]. This condition is recognized as a major public health concern worldwide, particularly among postmenopausal women [3], driven by the dual impact of estrogen deficiency and aging on bone health [4]. Estrogen plays a key role in regulating bone remodeling by inhibiting osteoclast-mediated bone resorption and supporting osteoblast function. Following menopause, the abrupt decline in estrogen levels disrupts this balance, resulting in accelerated bone loss [4,5]. Concurrently, aging contributes to a gradual decline in bone formation due to reduced osteoblast activity, impaired calcium absorption, and changes in bone microarchitecture [6,7]. This combination of hormonal and age-related factors synergistically increases the risk of osteoporotic fractures, especially those affecting the hip and spine, leading to significant morbidity, long-term disability, reduced quality of life, and increased mortality [8].

Osteoporosis affects an estimated 500 million people worldwide, with women facing a lifetime risk of osteoporotic fractures between 30–50%, compared to 15–30% in men [9]. According to the Global Burden of Disease Study 2019, low BMD was responsible for 438,000 deaths and 16.6 million disability-adjusted life years (DALYs) worldwide [1]. That same year, 178 million new fractures were recorded, contributing to 25.8 million years lived with disability (YLDs) [10]. As global populations continue to age, the global burden of osteoporosis is projected to rise sharply, creating significant challenges to healthcare systems. This issue is further compounded by the feminization of aging, as, on average, women live longer than men and are disproportionately affected by the disease.

In Brazil, osteoporosis also poses a significant and growing public health challenge. Recent national surveys estimate the prevalence of osteoporosis among Brazilian women over the age of 50 to range between 15% and 33%, depending on the diagnostic criteria and region studied [11]. The aging demographic profile of the Brazilian population, combined with lifestyle factors such as sedentary behavior, sub-optimal nutrition, and low vitamin D concentrations, has contributed to an increasing incidence of osteoporotic fractures. Hip fractures, in particular, have shown a rising trend in hospitalization rates, imposing significant costs on the Brazilian Unified Health System (SUS) and affecting patients’ functional independence and quality of life [8].

Given its significant clinical and socioeconomic impact, understanding the modifiable risk factors for osteoporosis is essential to guide public health interventions and preventive strategies. While traditional determinants, including age, sex, hormonal status, calcium and vitamin D intake, and physical activity, are well established [12], growing evidence suggests that environmental exposures to toxic metals, such as lead, cadmium, and arsenic, may also play a significant role in bone health and the development of osteoporosis [13,14,15], and this warrants further investigation.

Metals and metalloids are ubiquitous in the environment due to both natural and anthropogenic activities, and humans can be exposed through inhalation, ingestion, or dermal contact [16,17]. Several studies have investigated the influence of essential and toxic elements on bone health, revealing complex and sometimes contradictory effects. Essential trace elements, such as copper (Cu), manganese (Mn), selenium (Se), zinc (Zn), and cobalt (Co), are essential for maintaining normal physiological functions, including bone metabolism [18]. However, studies have shown that high levels of manganese (Mn) exposure may be associated with an increased risk of osteoporosis [19]. Zn has been shown to promote osteoblast proliferation and differentiation and bone matrix formation [20]. Selenium (Se), recognized for its antioxidant properties, may protect against oxidative stress [21,22], a factor that has been implicated in bone loss, and cobalt (Co) is involved in vitamin B12 metabolism, which may indirectly influence bone health [23]. However, both deficient and excessive intakes of these essential metals can have adverse effects [18,23].

Numerous epidemiological studies have highlighted the detrimental effects of exposure to toxic metals, particularly cadmium (Cd) and lead (Pb), on BMD and fracture risk [24,25,26]. Cd has been consistently linked to adverse skeletal outcomes. In a recent systematic review and meta-analysis conducted by our group, we demonstrated that even low-level environmental Cd exposure is linked to an increased risk of osteoporosis in postmenopausal women [27]. Similarly, low-level Pb exposure has been implicated in bone demineralization and disturbances in calcium homeostasis [28]. The effects of mercury (Hg) on bone health are less well defined. Although human data are lacking, some studies link Hg exposure to altered calcium homeostasis and increased osteoclast activity [29], while others report inconsistent findings [30]. Furthermore, elements such as antimony (Sb) and thallium (Tl) have been shown to induce oxidative stress [31,32,33], a process detrimental to bone integrity. However, their specific roles and underlying mechanisms in bone metabolism remain poorly understood and warrant further investigation.

Other less common elements, such as lithium (Li) and strontium (Sr), present more complex or even contradictory effects, depending on the exposure level and the population studied [34]. Lithium has been suggested to promote bone formation through the activation of the Wnt/β-catenin signaling pathway [35,36], a critical signaling pathway in osteogenesis [37], while Sr, although used therapeutically in osteoporosis management, may impair normal bone mineralization when present at high environmental levels [18,38,39]. Uranium (U) and cesium (Cs) are radioactive elements that may also interfere with calcium homeostasis in bone [40,41,42], though their effects in human populations remain poorly understood.

This cohort study seeks to address existing knowledge gaps by investigating the associations between exposure to a broad range of metals and metalloids—including aluminum (Al), barium (Ba), Cd, Co, Cs, Cu, Hg, Li, Mn, molybdenum (Mo), nickel (Ni), Pb, rubidium (Rb), Sb, Se, tin (Sn), Sr, Tl, U, and Zn—and BMD in postmenopausal women. By biomonitoring urinary metal(loid) levels, this study offers novel insights into how environmental exposure to these metals may affect bone health and contribute to osteoporosis risk.

## 2. Methods

### 2.1. Study Design and Population

We conducted a cross-sectional study using a cohort of 380 postmenopausal women. Ethical approval for the study was granted by the Research Ethics Committees of the State University of Western Paraná (Unioeste), Plataforma Brasil (approval number: 2.636.746), and Fernando Pessoa University in Porto, Portugal. Prior to participation, all individuals provided written informed consent.

Data collection spanned from March 2022 to February 2024. Participants were recruited through announcements disseminated via social media platforms, universities, medical clinics, health centers, hospitals, and both regional and municipal health departments. The inclusion criteria specified women between 50 and 70 years of age who were postmenopausal, defined retrospectively as experiencing at least 12 consecutive months without menstruation, and who had been residing in the study region for a minimum of 10 years. Exclusion criteria included the presence of serious active medical conditions such as advanced hepatic or renal insufficiency and cancer, a medically confirmed diagnosis of secondary osteoporosis—including but not limited to hyperparathyroidism, malignancies, or chronic corticosteroid therapy—as well as any history of occupational exposure to metals through industrial employment.

After obtaining informed consent, participants completed a structured questionnaire designed to collect data on osteoporosis risk factors, potential dietary and occupational metal exposures, as well as relevant sociodemographic and health-related variables. Prolonged bed rest was defined as a duration of 28 days or more, while insufficient physical activity was classified as engaging in less than 30 min of exercise per day. Following the questionnaire, trained research staff measured each participant’s body weight and height, from which body mass index (BMI) was calculated. The research team then coordinated the scheduling of urine sample collection and bone mineral density (BMD) assessments for all participants.

### 2.2. BMD Measurement

Bone mineral density (BMD) assessment was conducted for all participants using dual-energy X-ray absorptiometry (DEXA). The examinations were performed within the same radiology department utilizing the HOLOGIC Horizon-A model device. Measurements were obtained at standardized anatomical locations, specifically the lumbar spine, femoral neck, and total hip. The classification of bone health status followed the guidelines established by the World Health Organization (WHO). According to these criteria, osteoporosis is diagnosed when BMD is reduced by 2.5 SD or more at any of the evaluated bone sites.

### 2.3. Measurement of Urinary Metal(loid) Concentrations

First-morning urine samples were collected in metal-free tubes and stored at −20 °C until analysis. Urinary metal and metalloid concentrations were performed using inductively coupled plasma mass spectrometry (ICP-MS), equipped with a quadrupole ion deflector (NexION^®^ 2000, PerkinElmer, Shelton, CT, USA) and operated with high-purity argon gas (99.999%, Air Liquide, São Paulo, Brazil), following the analytical protocol previously established in the laboratory [43]. All reagents employed were of analytical grade (Sigma-Aldrich, St. Louis, MO, USA). Nitric acid (HNO_3_) used in sample preparation was further purified through sub-boiling distillation in a quartz distillation apparatus (Kürner Analysentechnik).

For each element analyzed, calibration curves with matrix matching were constructed using standard solutions ranging from 0 to 200 µg/L, prepared with a diluent composed of 0.5% HNO_3_ and 0.01% Triton X-100. Method accuracy and precision were evaluated using certified reference urine samples provided by the Institut National de Santé Publique du Québec (INSPQ) (QM-U-Q1509, Quebec, Canada). A 200 µL aliquot of each urine sample was diluted to a final volume of 5 mL with the prepared diluent, and all measurements were conducted in triplicate. Urinary element concentrations falling below the limit of detection (LOD) were estimated by assigning a value equal to the LOD divided by the square root of two (LOD/√2).

Urinary creatinine concentrations were determined using the alkaline picrate method on the Atellica^®^ CH analyzer (Siemens, São Paulo, Brazil). Metal and metalloid concentrations were then normalized to creatinine concentrations in urine and expressed as µg/g creatinine.

All procedures were performed at the Analytical and Systems Toxicology Laboratory (ASTox), Ribeirão Preto School of Pharmaceutical Sciences, University of São Paulo.

### 2.4. Statistical Analysis

Categorical variables were summarized as absolute and relative frequencies (n, %) and compared using the chi-square test or Fisher’s exact test, as appropriate. Quantitative variables were presented as medians with interquartile ranges (IQRs; Q1–Q3) and compared between groups using the Mann–Whitney U test due to non-normal distributions.

Associations between key variables were assessed using Spearman’s rank or Pearson correlation coefficients, with ln-transformation applied when necessary. A heatmap based on Spearman’s correlation values (selected due to non-normality) was used to visualize relationships among urinary concentrations of the analyzed elements.

Multiple linear regression with stepwise selection was used to identify independent variables associated with BMD; variables were ln-transformed when needed to meet model assumptions. Additionally, binary logistic regression was performed to evaluate the association between potential predictors and osteoporosis risk. Odds ratios (ORs) were adjusted for age, BMI, duration of menopause, smoking status, and prolonged bed rest.

All analyses were performed using IBM SPSS Statistics version 30.0. A two-tailed *p*-value < 0.05 was considered statistically significant.

## 3. Results

### 3.1. Characteristics of the Study Population

The sociodemographic characteristics, health habits, and clinical data of the postmenopausal women included in this study are summarized in Table 1. The median age of the 380 participants was 60 years. The median BMI was 27 kg/m^2^, with 75% of the women presenting a BMI above the upper-normal cutoff of 25 kg/m^2^. The majority of participants (93.7%) reported never having smoked or being former smokers, and only one woman reported current alcohol consumption. The overall prevalence of osteoporosis in the study population was 19.2%.

Table 1 also shows a comparison of these variables between women with and without a diagnosis of osteoporosis. Statistically significant differences were observed between the two groups in terms of age, BMI, menopausal duration, and calcium intake. Specifically, women diagnosed with osteoporosis were slightly older, had a lower BMI, experienced a longer postmenopausal period, and reported higher calcium intake. Additionally, a significantly higher proportion of women with osteoporosis reported using antiresorptive medication (*p* < 0.001).

As expected, bone mineral density (BMD) and T-scores at the lumbar spine, femoral neck, and total hip were significantly lower in women with osteoporosis compared to those without osteoporosis (*p* < 0.001).

### 3.2. Urinary Concentrations of Metals and Metalloids in Brazilian Postmenopausal Women

Table 2 depicts the urinary concentrations of 20 metals and metalloids for the entire study cohort, as well as separately for women with and without an osteoporosis diagnosis. After adjusting for age, BMI, duration of menopause, smoking status, and prolonged bed rest, statistically significant differences in median urinary concentrations were identified between the two groups for Cd, Mn, Pb, Sb, Sn, and Zn. Specifically, women with osteoporosis had higher median urinary concentrations of Cd (0.38 µg/g creatinine vs. 0.30 µg/g creatinine, *p* = 0.012), Mn (7.0 µg/g creatinine vs. 4.1 µg/g creatinine, *p* = 0.014), Pb (4.8 µg/g creatinine vs. 3.7 µg/g creatinine, *p* = 0.020), Sb (0.27 µg/g creatinine vs. 0.17 µg/g creatinine, *p* = 0.015), Sn (0.70 µg/g creatinine vs. 0.49 µg/g creatinine, *p* = 0.046), and Zn (860 µg/g creatinine vs. 777 µg/g creatinine, *p* = 0.004) compared to women without osteoporosis.

### 3.3. Intercorrelations Between Metal(loid) Concentrations in Urine

**Figure 1:**
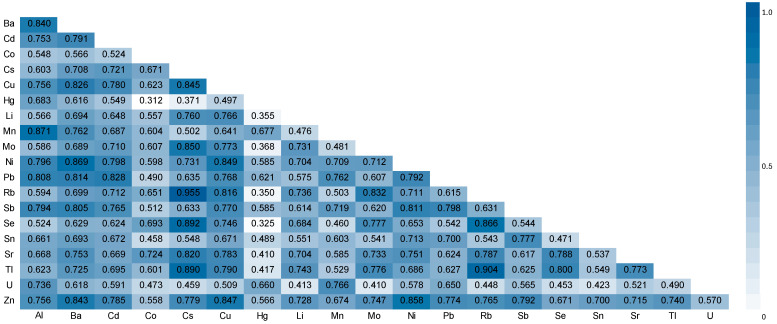
Heatmap plot showing correlation between the elements in urine. Spearman’s rank correlation coefficients (R).

Figure 1 presents the heatmap generated from Spearman correlation coefficients calculated for all measured metal(loid) concentrations in urine of the study population. Notably, several strong positive correlations were observed between creatinine-adjusted element concentrations. The strongest correlations in decreasing order were between Rb and Cs (0.955, *p* < 0.01), Tl and Rb (0.904, *p* < 0.01), Se and Cs (0.892, *p* < 0.01), Tl and Cs (0.890, *p* < 0.01), Mn and Al (0.871, *p* < 0.01), Ni and Ba (0.869, *p* < 0.01), Se and Rb (0.866, *p* < 0.01), Zn and Ni (0.858, *p* < 0.01), Mo and Cs (0.850, *p* < 0.01), Ni and Cu (0.849, *p* < 0.01), Zn and Cu (0.847, *p* < 0.01), Cu and Cs (0.845, *p* < 0.01), Zn and Ba (0.843, *p* < 0.01), Ba and Al (0.840, *p* < 0.01), Rb and Mo (0.832, *p* < 0.01), Zn and Cu (0.828, *p* < 0.01), Cu and Ba (0.826, *p* < 0.01), Sr and Cs (0.820, *p* < 0.01), Rb and Cu (0.816, *p* < 0.01), Pb and Ba (0.814, *p* < 0.01), Sb and Ni (0.811, *p* < 0.01), Pb and Al (0.808, *p* < 0.01), and Sb and Ba (0.805, *p* < 0.01).

### 3.4. Association of Studied Variables (Clinical and Analytical) with BMD

The statistically significant associations obtained between BMD at the lumbar spine, femoral neck, and total hip and age, BMI, menopause length, and urinary metal(loid) concentrations are presented in Table 3. Age and menopause length showed negative correlations with BMD at all three sites, while BMI showed positive correlations. Among the metals and metalloids, weak but statistically significant negative correlations were observed between BMD and urinary concentrations of Al (lumbar spine, total hip), Cd (lumbar spine, total hip), Hg (total hip), Mn (lumbar spine), Sb (lumbar spine, total hip), and U (lumbar spine, femoral neck, total hip).

Given that lumbar spine was the bone site where most cases of osteoporosis were diagnosed, we sought to determine the primary determinants of BMD at this location. Multiple linear regression analysis identified ln-transformed BMI as the strongest positive predictor of lumbar spine BMD, while ln-transformed menopause length, smoking, and prolonged bed rest were significant negative predictors (Table 4). No metals or metalloids were included as significant predictors of lumbar spine BMD in this model.

### 3.5. Association Between Urinary Metal(loid) Concentrations and Osteoporosis Risk

**Figure 2:**
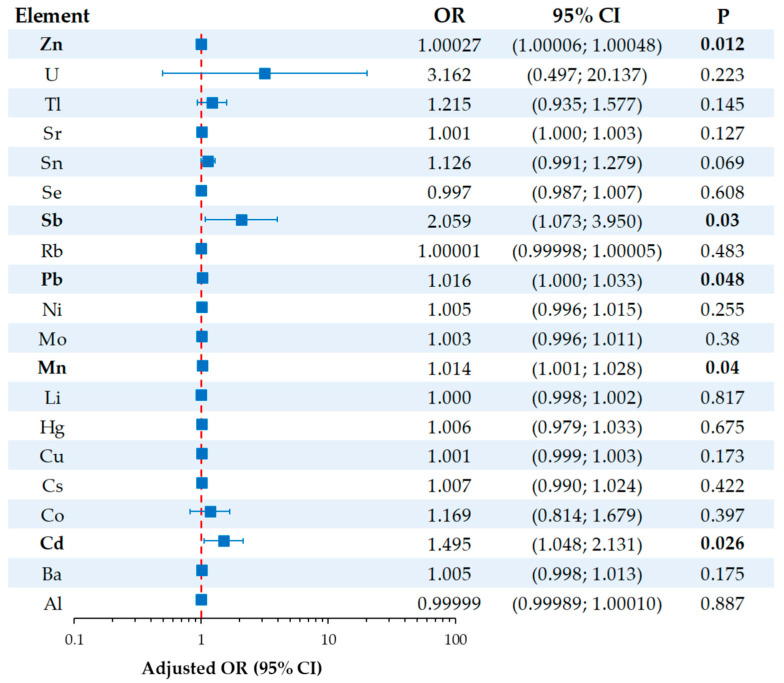
Forest plot showing the independent adjusted odds ratios (aORs) for osteoporosis outcomes associated with metal(loid) exposure. ORs were adjusted for age, BMI, length of menopause, smoking, and prolonged bed rest. Statistically significant *p*-values are shown in bold.

Independent adjusted odds ratios (aORs) for osteoporosis outcome associated with ln-transformed urinary metal and metalloid variables are presented in Figure 2. After adjusting for age, BMI, length of menopause, smoking, and prolonged bed rest, urinary Cd (aOR = 1.495, 95% CI: 1.048; 2.131, *p* = 0.026), Mn (aOR = 1.014, 95% CI: 1.001; 1.028, *p* = 0.040), Sb (aOR = 2.059, 95% CI: 1.073; 3.950, *p* = 0.030), and Zn (aOR = 1.00027, 95% CI: 1.00006; 1.00048, *p* = 0.012) were significantly associated with osteoporosis outcome. However, only Cd and Sb showed clinically meaningful associations, as the odds ratios for Mn and Zn were very close to 1.

Given that Cd and Sb demonstrated the strongest and most clinically meaningful associations with increased osteoporosis risk, we further analyzed their combined effects. Women were categorized based on whether their urinary concentrations of both elements were below or at/above the 90th percentile thresholds (1.079 μg/g creatinine for cadmium and 0.729 μg/g creatinine for antimony; see Supplementary Table S1). Notably, women with elevated urinary concentrations exhibited significantly lower BMD at all measured bone sites and a higher prevalence of osteoporosis (44.4% vs. 18.0%; *p* = 0.011).

## 4. Discussion

This cross-sectional study investigated the association between urinary concentrations of 20 metal(loid)s and osteoporosis in a cohort of postmenopausal Brazilian women. Several significant associations were identified. Consistent with established risk factors for osteoporosis, women diagnosed with the disease were generally older, had a lower BMI, and experienced a longer time since menopause onset. Moreover, increased urinary concentrations of aluminum, cadmium, mercury, manganese, antimony, and uranium were correlated with decreased BMD. Notably, this study is the first to demonstrate a significant association between elevated urinary concentrations of both cadmium and antimony and an increased risk of postmenopausal osteoporosis, even after adjusting for potential confounders. In light of the increasing environmental burden of these elements from industrial, mining, and vehicular sources [44], our findings reinforce the importance of further human and environmental monitoring studies.

Biomonitoring studies are crucial for assessing population-level exposure to environmental metals and metalloids, identifying vulnerable groups, and informing risk mitigation efforts. Beyond characterizing exposure–outcome relationships, biomonitoring provides an essential tool for evaluating the effectiveness of regulatory policies over time [45,46]. Urine is widely recognized as a suitable biological matrix for such assessments, owing to its non-invasive collection and its reliability for specific metals. For elements such as cadmium, cobalt, cesium, molybdenum, nickel, antimony, strontium, thallium, and uranium, urinary concentrations serve as robust biomarkers of exposure due to their relatively stable excretion profiles [47]. In contrast, for metals such as lead, mercury, manganese, aluminum, copper, and zinc, urinary levels are considered less reliable indicators of exposure [48]. This limitation arises from alternative primary excretion routes (e.g., biliary or fecal pathways) or tight homeostatic regulation, which can obscure the relationship between exposure and urinary elimination.

In our study, urinary concentrations of two essential trace metals—manganese and zinc—and of four toxic metal(loid)s—cadmium, lead, tin, and antimony—were higher in osteoporotic women compared with non-osteoporotic controls (Table 2). Both Mn and Zn were reported to present a U-shaped curve in terms of their toxicity and effect on bone health. This means that, at low levels, they are essential and beneficial for bone formation and mineralization, whereas, at excessively high levels, they become toxic, leading to impaired bone metabolism and bone loss [18]. Therefore, the increased urinary excretion of Mn and Zn observed in the osteoporosis group may reflect heightened environmental exposure but could also result from osteoporosis-induced loss of essential trace elements, as previously described [49]. This loss may further exacerbate deficiencies that are critical for maintaining bone health. Conversely, the elevated urinary concentrations of Cd, Pb, Sn, and Sb are more likely attributable to increased environmental exposure to these toxic metals, which are known to interfere with bone remodeling and mineralization, thereby potentially contributing to osteoporosis risk [14]. Among them, in the present study only cadmium and antimony showed significant negative correlations with BMD (Table 3), implicating a potential role in bone deterioration. However, the observed correlation coefficients were modest, indicating weak predictive value when considered individually. This was corroborated by multiple linear regression analysis, which failed to identify any of the metals as independent predictors of lumbar spine BMD (Table 4).

Nevertheless, potential adverse effects may become more pronounced at higher exposure levels, as we recently demonstrated for Cd in Brazilian postmenopausal women [50], or in scenarios involving co-exposure to multiple metal(loid)s. In our study, women with urinary cadmium and antimony concentrations above the 90th percentile exhibited a significantly higher prevalence of postmenopausal osteoporosis (Supplementary Table S1). This finding underscores the possible additive or synergistic effects of Cd and Sb on bone degradation, in agreement with previous reports on the combined impact of metal mixtures on BMD reduction [51]. Notably, both cadmium and antimony tend to accumulate in soil and crops due to industrial emissions and agricultural practices [52,53], increasing the likelihood of concurrent human exposure through dietary intake or shared environmental sources. This co-exposure scenario is further supported by the strong positive correlation observed between urinary concentrations of these metals in our cohort (*p* = 0.765; Figure 1).

Substantial epidemiological evidence indicates that chronic exposure to cadmium is associated with impaired bone metabolism, reduced BMD, and increased risk of osteopenia and osteoporosis [14,27,54,55]. Cadmium exerts its deleterious effects through multiple mechanisms, including renal dysfunction, disruption of calcium homeostasis, oxidative stress, and inflammation. Specifically, cadmium inhibits osteoblast differentiation while enhancing osteoclast activity, partly through interference with Wnt/β-catenin signaling and upregulation of RANKL expression [15,56]. Cadmium-induced oxidative stress activates NF-κB and increases proinflammatory cytokines such as IL-6 and TNF-α, further contributing to bone resorption [57]. Additionally, cadmium disrupts calcium metabolism by impairing gastrointestinal absorption, renal reabsorption, and vitamin D receptor-mediated synthesis of 1,25-dihydroxyvitamin D [58]. Proteomic and metabolomic analyses have also revealed cadmium-related alterations in osteogenic gene expression, bone matrix protein production, and mineralization pathways, further implicating cadmium in skeletal fragility [59]. In contrast, data on antimony remain limited. Given its chemical similarity to arsenic, a recognized osteotoxicant, antimony may exert analogous effects, including oxidative stress induction and disruption of osteoblast function [33]. Some experimental studies have linked high antimony levels to redox imbalance and increased bone loss [60]. However, the evidence remains sparse and inconsistent, highlighting the need for additional in vivo and in vitro studies to elucidate antimony’s role in bone demineralization and osteoporosis risk.

The adjusted odds ratios provided deeper insight into the independent relationships between metals and metalloid exposure and osteoporosis risk (Figure 2). Antimony demonstrated the strongest association (aOR = 2.059), suggesting that higher urinary antimony concentrations are associated with more than a two-fold increase in the odds of developing osteoporosis. Cadmium was also associated with an increased risk (aOR = 1.495), indicating a moderate but clinically meaningful effect. Statistically significant associations were observed for manganese and zinc as well; however, their effect sizes were minimal, as indicated by the low odds ratios. The significance for manganese and zinc likely reflects the large sample size and narrow confidence intervals rather than a meaningful biological impact.

To the best of our knowledge, only two studies to date have investigated the associations between urinary concentrations of multiple metallic elements and BMD loss or osteoporosis risk in older and/or postmenopausal women. The first study, based on data from the U.S. National Health and Nutrition Examination Survey (NHANES) 2005–2010, supports our findings by identifying urinary cadmium, along with arsenic and tungsten, as negatively associated with BMD [61]. A more recent analysis of NHANES 2017–2020 data found no statistically significant associations between BMD in women and any of the 11 examined metal(loid)s (Ba, Cd, Co, Cs, Mo, Mn, Pb, Sb, Sn, Tl, and W) after adjusting for potential confounders. However, a consistent negative trend was observed for all elements except manganese. This may reflect the impact of successful public health interventions that have substantially reduced environmental exposures in the U.S. population over time [62]. As a result, current exposure levels may be below the biological threshold needed to influence bone health. Notably, the NHANES 2017–2020 study reported a sex-specific effect of antimony: urinary antimony levels were negatively associated with femoral BMD in women but positively associated in men [63]. These findings, consistent with our data, suggest that antimony exposure may represent a sex-specific risk factor for bone loss in women. Further research is needed to confirm these associations and elucidate the biological mechanisms underlying antimony toxicity.

Although the observed associations underscore the potential role of chronic low-level metal exposure as a modifiable risk factor for postmenopausal osteoporosis, the following limitations warrant consideration. First, the cross-sectional design precludes causal inference, and reverse causation, whereby osteoporosis influence on metal and metalloid excretion cannot be completely excluded. Second, the study population consists of postmenopausal women from a specific region of Brazil, which may limit the generalizability of the results. Third, although creatinine adjustment was applied to ensure comparability with previous studies, it may introduce bias due to variability in muscle mass [64]. This limitation should be acknowledged when interpreting the results. Finally, we did not assess dietary intake or other potential sources of metal and metalloid exposure (e.g., traffic emissions), which could also influence the observed associations.

Future longitudinal studies are essential to confirm these associations and to elucidate the underlying mechanisms. Additionally, identifying the primary sources of exposure would further support the development of effective prevention strategies.

## 5. Conclusions

This study adds to the growing body of evidence that environmental exposure to metals and metalloids is significantly associated with reduced BMD and increased odds of osteoporosis in postmenopausal women. Antimony demonstrated the strongest association with osteoporosis risk, while cadmium was linked to a moderate yet clinically relevant risk. While causality cannot be established, the findings highlight the need to address environmental metal exposure as part of broader strategies to preserve bone health. Future longitudinal and mechanistic studies are essential to confirm these associations and to inform risk assessment and mitigation efforts.

## Figures and Tables

**Table 1:** Clinical and sociodemographic characteristics of participants, presented overall and according to osteoporosis diagnosis.

Variables	Overall n = 380	No Osteoporosis n = 307	With Osteoporosis n = 73	*p*-Value ^1^
Age (years)	60.0 (56.0; 65.8)	60.0 (56.0; 65.0)	62.0 (59.0; 66.0)	**0.011**
BMI (kg/m^2^)	27.0 (24.4; 30.2)	27.6 (24.8; 30.8)	26.1 (23.4; 28.1)	**<0.001**
Length of menopause (years)	13.0 (7.3; 19.0)	12.0 (7.0; 18.0)	16.0 (10.5; 21.5)	**0.002**
Prior fracture (yes)	109 (28.7%)	84 (27.4%)	25 (34.2%)	0.252
Arthritis (yes)	72 (18.9%)	62 (20.2%)	10 (13.7%)	0.246
Vitamin D intake (yes)	183 (48.2%)	149 (48.5%)	34 (46.4%)	0.795
Corticoids (yes)	88 (23.2%)	70 (22.8%)	18 (24.7%)	0.758
Prolonged bed rest (yes)	28 (7.4%)	21 (6.8%)	7 (9.6%)	0.454
No exercise (yes)	135 (35.5%)	112 (36.5%)	23 (31.5%)	0.497
Calcium intake (yes)	100 (26.3%)	68 (22.1%)	32 (43.8%)	**<0.001**
Alcohol intake (yes)	1 (0.3%)	1 (0.3%)	0 (0%)	1.000
Smoking (yes)	24 (6.3%)	16 (5.2%)	8 (11.0%)	0.333
Antiresorptive medications				**<0.001**
Bisphosphonates	10 (2.6%)	4 (1.3%)	6 (8.2%)	
Bisphosphonates and HRT	1 (0.3%)	0 (0%)	1 (1.4%)	
HRT	45 (11.8%)	33 (10.7%)	12 (16.4%)	
No	324 (85.3%)	270 (87.9%)	54 (74.0%)	
Lumbar spine				
BMD (g/cm^2^)	0.92 (0.82; 1.06)	0.97 (0.88; 1.08)	0.74 (0.70; 0.77)	**<0.001**
T-score	−1.10 (−2.00; 0.10)	−0.70 (−1.50; 0.40)	−2.80 (−3.10; −2.50)	**<0.001**
Diagnosis of osteoporosis	61 (16.1%)	0 (0%)	61 (83.6%)	**<0.001**
Femoral neck				
BMD (g/cm^2^)	0.73 (0.65; 0.83)	0.75 (0.68; 0.86)	0.63 (0.56; 0.71)	**<0.001**
T-score	−1.10 (−1.78; −0.20)	−0.90 (−1.50; 0.10)	−2.00 (−2.65; −1.35)	**<0.001**
Diagnosis of osteoporosis	25 (6.6%)	0 (0%)	25 (34.2%)	**<0.001**
Total hip				
BMD (g/cm^2^)	0.87 (0.78; 0.96)	0.90 (0.81; 0.98)	0.74 (0.69; 0.84)	**<0.001**
T-score	−0.60 (−1.30; 0.10)	−0.40 (−1.00; 0.30)	−1.60 (−2.05; −0.90)	**<0.001**
Diagnosis of osteoporosis	10 (2.6%)	0 (0%)	10 (13.7%)	**<0.001**
Urinary creatinine (mg/dL)	52.7 (29.0; 90.9)	52.8 (31.1; 93.1)	52.5 (22.2; 85.4)	0.090

Results are presented as median (first quartile; third quartile), unless otherwise indicated; ^1^ Mann–Whitney test; Statistically significant differences are shown in bold. Abbreviations: BMD, Bone mineral density; BMI, Body mass index; HRT, Hormone replacement therapy. Statistically significant *p*-values are shown in bold.

**Table 2:** Urinary concentrations (expressed in μg/g creatinine) of metals and metalloids, presented overall and according to osteoporosis diagnosis.

Elements (µg/g creat)	Overall n = 380	No Osteoporosis n = 307	With Osteoporosis n = 73	*p*-Value ^1^
Al	217 (108; 448)	202 (106; 425)	340 (129; 638)	0.273
Ba	16.5 (8.3; 29.0)	15.4 (8.0; 27.8)	19.7 (9.0; 36.8)	0.127
Cd	0.30 (0.15; 0.55)	0.30 (0.14; 0.49)	0.38 (0.16; 0.71)	**0.012**
Co	0.23 (0.09; 0.46)	0.22 (0.08; 0.46)	0.26 (0.13; 0.66)	0.543
Cs	7.9 (4.0; 14.0)	7.7 (4.0; 13.5)	8.2 (4.6; 15.5)	0.419
Cu	93.6 (53.1; 172.9)	92.0 (52.9; 169.9)	98.4 (57.6; 184.7)	0.148
Hg	0.99 (0.41; 2.07)	0.97 (0.40; 1.93)	1.03 (0.55; 2.91)	0.501
Li	6.9 (3.2; 14.9)	6.7 (3.2; 14.8)	8.5 (3.9; 17.0)	0.578
Mn	4.4 (1.7; 9.7)	4.1 (1.7; 8.8)	7.0 (2.4; 15.7)	**0.014**
Mo	17.0 (8.6; 33.4)	17.0 (8.6; 32.3)	16.5 (9.3; 37.7)	0.378
Ni	16.0 (8.1; 30.9)	15.8 (8.0; 28.9)	17.5 (9.1; 35.9)	0.219
Pb	4.0 (2.1; 8.1)	3.7 (2.1; 7.4)	4.8 (2.7; 11.7)	**0.020**
Rb	3105 (1567; 5742)	3026 (1557; 5627)	3636 (1649; 6220)	0.570
Sb	0.19 (0.10; 0.39)	0.17 (0.10; 0.36)	0.27 (0.13; 0.52)	**0.015**
Se	11.7 (5.9; 24.4)	12.0 (6.0; 25.6)	10.8 (5.5; 22.9)	0.439
Sn	0.52 (0.23; 1.09)	0.49 (0.22; 0.92)	0.70 (0.24; 1.86)	**0.046**
Sr	76.4 (40.7; 179.3)	75.8 (38.5; 178.7)	87.9 (51.3; 215.3)	0.100
Tl	0.22 (0.11; 0.46)	0.21 (0.11; 0.45)	0.26 (0.12; 0.54)	0.076
U	0.017 (0.005; 0.041)	0.016 (0.005; 0.038)	0.021 (0.008; 0.056)	0.109
Zn	808 (458; 1548)	777 (460; 1510)	860 (456; 1882)	**0.004**

Results are presented as median (first quartile; third quartile); ^1^ Generalized Linear Model (GLM) of the ln-transformed elements adjusted for age (years), BMI (Kg/m^2^), length of menopause (years), smoking, and prolonged bed rest. Statistically significant differences are shown in bold. Abbreviations: Al, aluminum; Ba, barium; Cd, cadmium; Co, cobalt; Cs, cesium; Cu, copper; Hg, mercury; Li, lithium; Mn, manganese; Mo, molybdenum; Ni, nickel; Pb, lead; Rb, rubidium; Sb, antimony; Se, selenium; Sn, tin; Sr, strontium; Tl, thallium; U, uranium; Zn, zinc. Statistically significant *p*-values are shown in bold.

**Table 3:** Statistically significant associations between ln-transformed bone mineral density (BMD) at the lumbar spine, femoral neck, and total hip and ln-transformed age, BMI, menopause length, and urinary metal(loid) concentrations.

		BMD Lumbar Spine	BMD Femoral Neck	BMD Total Hip
BMD Lumbar Spine	r	1	0.525	0.697
	*p*		**<** **0.001**	**<** **0.001**
BMD Femoral Neck	r	0.525	1	0.743
	*p*	**<** **0.001**		**<** **0.001**
BMD Total Hip	r	0.697	0.743	1
	*p*	**<** **0.001**	**<** **0.001**	
Age	r	−0.182	−0.256	−0.278
	*p*	**<** **0.001**	**<** **0.001**	**<** **0.001**
BMI	r	0.301	0.311	0.456
	*p*	**<** **0.001**	**<** **0.001**	**<** **0.001**
Menopause Length	r	−0.247	−0.220	−0.271
	*p*	**<** **0.001**	**<** **0.001**	**<** **0.001**
Al	r	−0.112	−0.062	−0.104
	*p*	**0.030**	0.227	**0.042**
Cd	r	−0.102	−0.064	−0.128
	*p*	**0.048**	0.215	**0.013**
Hg	r	−0.084	−0.062	−0.128
	*p*	0.101	0.226	**0.012**
Mn	r	−0.128	−0.072	−0.097
	*p*	**0.012**	0.159	0.058
Sb	r	−0.106	−0.068	−0.104
	*p*	**0.039**	0.186	**0.044**
U	r	−0.107	−0.103	−0.116
	*p*	**0.036**	**0.044**	**0.023**

r, Pearson correlation coefficient; Abbreviations: Al, aluminum; BMD, bone mineral density; BMI, body mass index; Cd, cadmium; Hg, mercury; Mn, manganese; Sb, antimony; U, uranium. Statistically significant *p*-values are shown in bold.

**Table 4:** Main variables associated with bone mineral density at the lumbar spine, by multiple linear regression analysis.

Dependent Variable	Model	Unstandardized Coefficients		Standardized Coefficients	t	*p*-Value
		Beta (95% CI)	Std. Error	Beta		
Ln BMD	(Constant)	−0.956 (−1.277; −0.636)	0.163		−5.863	**<0.001**
	Ln BMI	0.310 (0.214; 0.405)	0.049	0.299	6.366	**<0.001**
	Ln menopause Length	−0.052 (−0.072; −0.032)	0.010	−0.242	−5.140	**<0.001**
	Smoking	−0.024 (−0.045; −0.004)	0.010	−0.111	−2.358	**0.019**
	Prolonged bed rest	−0.067 (−0.129; −0.004)	0.032	−0.099	−2.096	**0.037**

Abbreviations: BMD, bone mineral density; BMI, body mass index. Statistically significant *p*-values are shown in bold.

## Data Availability

The data presented in this study are available on request from the corresponding author due to privacy restrictions.

## Supplementary Materials

The following supporting information can be downloaded at: https://www.mdpi.com/article/10.3390/toxics13060489/s1, Table S1: Comparison of participants with urinary concentrations of both Cd and Sb below and at or above the 90th percentile (P90; 1.079 and 0.729 μg/g creatinine for Cd and Sb, respectively).

## Abbreviations

The following abbreviations are used in this manuscript:

aOR	Adjusted odds ratio
BMI	Body mass index
DALYs	Disability-adjusted life years
DEXA	Dual-energy X-ray absorptiometry
ICP-MS	Inductively coupled plasma–mass spectrometry
HRT	Hormone replacement therapy
NHANES	U.S. National Health and Nutrition Examination Survey
WHO	World Health Organization
YLDs	Years lived with disability
