# Supplementary material for: Association of Urinary Cadmium and Antimony with Osteoporosis Risk in Postmenopausal Brazilian Women: Insights from a 20 Metal(loid) Biomonitoring Study
Source: Toxics. 2025 Jun 10;13(6):489. doi: 10.3390/toxics13060489 (PMC12197107; doi:10.3390/toxics13060489)
Supplement: Supplementary file 1 [file toxics-13-00489-s001.zip › toxics-3651354-supplementary.pdf]

Supplementary Table S1. Comparison of participants with urinary concentrations of both Cd and Sb below and at or above the 90th percentile (P90; 1.079 and 0.729 μg/g creatinine for Cd and Sb, respectively).

**Variables**	**Overall** n = 380	**Cd and Sb < P90** n = 362	**Cd and Sb ≥ P90** n = 18	***p*-Value**[1]
Age (years)	60.0 (56.0; 65.8)	60.0 (56.0; 65.3)	63.5 (58.5; 66.0)	0.182
BMI (kg/m^2)	27.0 (24.4; 30.2)	27.2 (24.8; 30.4)	24.3 (20.9; 26.6)	**0.003**
Length of menopause (years)	13.0 (7.3; 19.0)	13.0 (7.0; 19.0)	16.0 (9.3; 21.0)	0.310
Prolonged bed rest	28 (7.4%)	27 (7.5%)	1 (5.6%)	1.000
Smoking	24 (6.3%)	24 (6.6%)	0 (0%)	0.266
Urinary Cd (μg/g creat)	0.30 (0.15; 0.55)	0.29 (0.14; 0.49)	1.44 (1.18; 2.98)	**<0.001**
Urinary Sb (μg/g creat)	0.19 (0.10; 0.39)	0.18 (0.10; 0.35)	1.17 (0.86; 1.58)	**<0.001**
Lumbar BMD (g/cm^2)	0.92 (0.82; 1.06)	0.93 (0.83; 1.06)	0.81 (0.72; 0.92)	**0.005***
Femoral BMD (g/cm^2)	0.73 (0.65; 0.83)	0.74 (0.66; 0.83)	0.64 (0.59; 0.68)	**<0.001***
Total hip BMD (g/cm^2)	0.87 (0.78; 0.96)	0.88 (0.79; 0.96)	0.76 (0.69; 0.84)	**<0.001***
Osteoporosis (Yes)	73 (19.2%)	65 (18.0%)	8 (44.4%)	**0.011**

Results are presented as median (first quartile; third quartile); [1]Mann-Whitney test; Statistically significant differences are shown in bold. Abbreviations: BMD, Bone mineral density; BMI, Body mass index; Cd, Cadmium; Sb, antimony. *, Statistical significance was kept after adjustment for BMI (P<0.05). Statistically significant *p*-values are shown in bold.
